# The nanocomposites designs of phytomolecules from medicinal and aromatic plants: promising anticancer-antiviral applications

**DOI:** 10.1186/s43088-022-00198-z

**Published:** 2022-01-29

**Authors:** Ayse Kaplan

**Affiliations:** Department of Molecular Biology, Faculty of Science, Eskisehir Technical University, Eskisehir, Turkey

**Keywords:** Saffron, Lavender, Clove, Red beet, Phytonanocomposite, Anticancer-antiviral activity

## Abstract

**Background:**

Nowadays, researchers are moving toward a herbal approach to cancer treatment because of the harmful effects of synthetic anti-tumor drugs. The evaluation of active compounds with plant origin may help in the remedy of human illnesses in the future. These active compounds have direct or indirect curative efficacies on difficult to cure diseases such as cancer. Investigation of nanoforms of these active compounds is one of the curious topics of the scientific community.

**Main body:**

Saffron and its components obtained from *Crocus sativa*, essential oils obtained from lavender, *Syzygium aromaticum* called cloves and *Beta vulgaris* are known for their anticancer effects. Nano-drugs are designed to increase the anticancer activity of plant-derived drugs. Herbal extracts operate very great in the production of nanoparticles. The aim is to ensure that only the nano-drug is delivered to the tumor site. Furthermore, nanoparticles have hazardous effects when analyzed at elevated doses, but this issue can be doped together with plant extracts.

**Short conclusions:**

The nanocomposites (graphene oxide, solid lipid nano and nanoemulsion) of phytomolecules obtained from saffron, clove, lavender and red beet may be effective in minimizing these toxic effects. In the near future, detecting the anticancer molecular mechanisms of these naturally derived compounds and nanocomposites could contribute to further cancer research. Apart from these, these compounds and its nanocomposites could have antiviral effects against today's threat covid-19 virus. Consequently, more promising anticancer and antiviral agents would be discovered.

**Graphical abstract:**

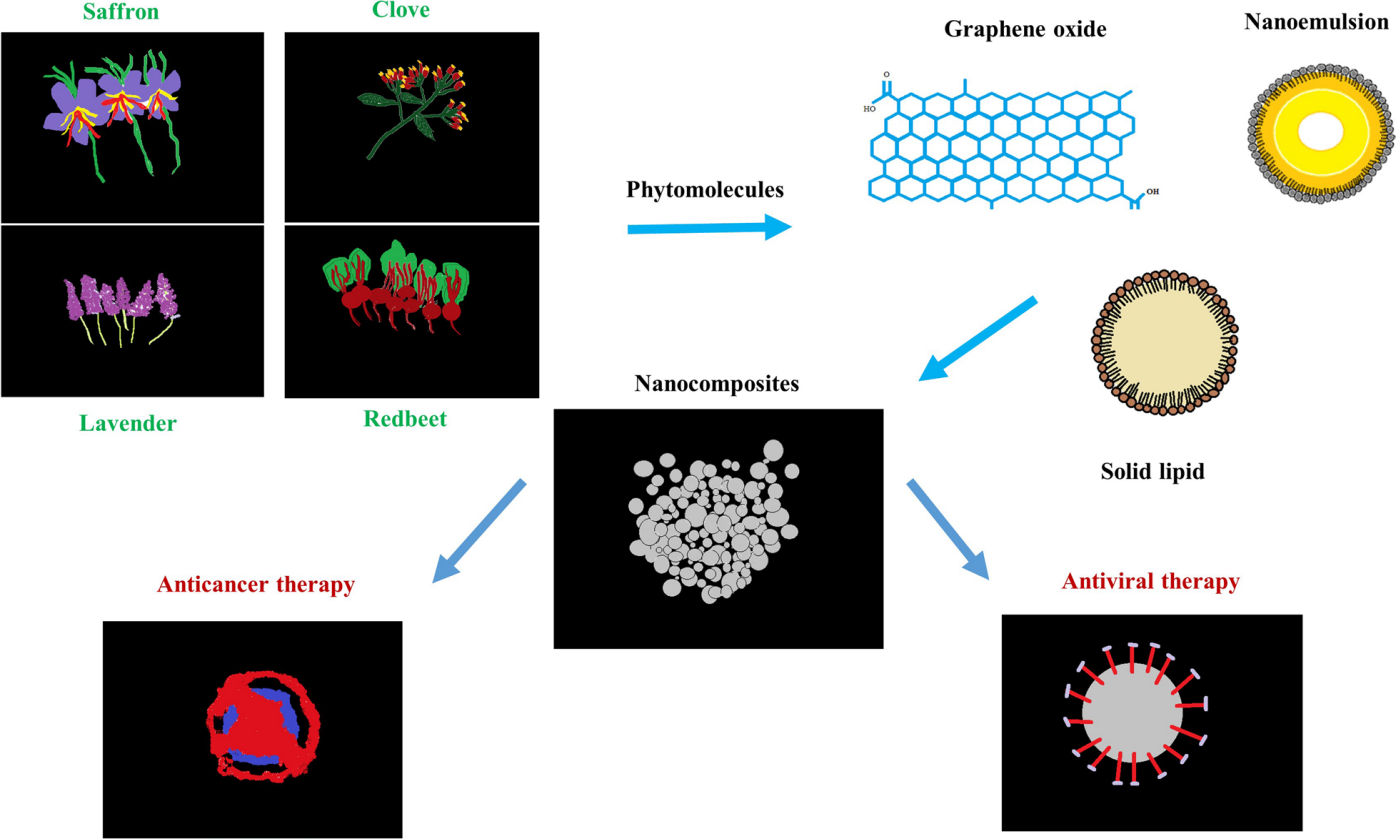

## Background

Human communities have been in close contact with their environment since their existence and have used their environmental components to acquire nutrient and medicament. The practices of herbs in obtaining nutrient and medicament have been carried out by test and error and over time, they could meet their needs with the human environment. The knowledge about medicinal plants has been progressively transferred over the years, and human knowledge has been progressively completed, with the genesis of cultures and constitution of more facilities from generation to generation. Medicinal herbs are nearly used as a medicinal source all cultures. The assessments of bioactive components can help cure many ailments in the future [42].

When the improvements in the manufacture and use of medicinal and aromatic plants in the twentieth century are analyzed, the innovations, social and political changes brought about by the technology at the turn of the century have given rise to the fast utilization of plants as medicines. The syntheses of sulfa drugs in the 1930s and organic chemicals in the 1940s have encouraged the production of synthetic drugs in addition to medicinal plants. Economic and social changes following World War II, and new definitions about plants and treatments have caused a decrease in the utilization of plants with plant extracts till the end of 1970s in the western countries that modernized with industrial advances as a result of obtaining synthetic chemical drugs. While more than 40% of the medicines listed in the early twentieth century (mostly from unrefined) are of vegetable origin, this issue has dropped below 5% by the mid-1970s. In the 1980s and 1990s, more information about the health of the consumers, especially the interest observed in favor of herbal medicines in developed countries, the orientation toward organic and natural foods have brought medicinal and aromatic plants back to the agenda [12]. Researches have started on medicinal and aromatic plants in the 1980s and 1990s have brought about improvements in the manufacturing of plants, extraction of bioactive components and verification of medicinal practices [57].

Cosmetologists have formulated products containing numerous herbal extracts, such as *Aloe barbadensis*, *Celastrus paniculatus*, *Cyperus scariosus*, *Ginkgo biloba*, *Myrtus caryophyllus* and *Withania somnifera* [62]. Thus, medicinal plants have been in demand and their acceptance has gradually increased. The pieces of medicinal plants that are generally utilized in herbal therapy; different types of germs can be stem, leaf, fruit, flower or whole plant [89]. Active compounds derived from herbs have direct or indirect curative effects. These plant-derived active compounds have significantly remedied difficult to cure diseases such as cancer. These ingredients can also prohibit the advancement of some illness [95]. The importance and production of medicinal plants are increasing due to some harm to the body of synthetic drugs used in diseases. Phytotherapy has found a place worldwide and the global tendency of synthetic compounds has turned into a plant. Plant-based traditional medicines such as aspirin (from willow bark), digoxin, morphine (opium poppy) are currently based on the basis of plants that have clinical, pharmaceutical and chemical studies [42].

Today, targeted drug delivery is one of the hopeful remedies to handle these problems. The use of nanostructures as a drug carrier has attracted major interest as it can burden large amounts of chemotherapeutic agents and is also effectively and efficiently distributed the drug load to the tumor location. According to th literature, the utilization of nanostructures in targeted drug delivery has advances such as advanced specificity of the drug and therapeutic effect of treatment. The most common nanomaterials have formulated as drug carriers occur from lipid-based, polymeric-based, graphene-based nanomaterials [67]. Also, nanoemulsions are broadly utilized targeted drug delivery of various antitumor agents in pharmaceutical systems [41]. Phytomolecules are being investigated to cure numerous ailments conditions, such as diabetes, cardiovascular, cancer, microbial disease, and inflammatory activity. Although phytomolecules have unique benefits, such as fewer side effects, low-price, lower toxicity, and therapeutic potential, they have caused toxicity, and biocompatibility challenge of the phytomolecules. Therefore, nano-phytomedicines have an important effective in advanced drug and pharmaceutical production, with enormous success in delivery, targeting space, and drug release in the controlled drug delivery system [7].

## Main text

### The importance of medicinal and aromatic plants in the future

Medicinal plants have an important place as the main source in the basic health systems of local communities. The utilization of medicinal plants has a long history [10]. Although there are half a million plants worldwide, most plants have not been examined in the treatment of diseases. Scientists must be educated in advanced and conventional medicine in the utilization of herb composites. Finally, safety, correct dosage, period of cure, harmful effects, acute and chronic herbal standardization, as well as toxicities, medicines and natural products should be addressed [42].The National Cancer Institute (NCI) has scanned about 35,000 herbal species, which anticancer properties [88]. Cancer is one of the main reasons of death and disease in the world and the count of cases is calculated to be 21 million by 2030. In 2017, it has been calculated that only the USA has experienced almost 1,688,780 new cancer diagnostics and 600,920 cancer deaths [88]. It has been proved that these plant-derived active compounds significantly improve difficult to treat diseases such as cancer [95].

### Some plant-derived therapeutics with anticancer potential

Cancer kills millions of people worldwide every year [11]. Thus, it acts as a great impediment in the socio-economic progress of nations. Cancer is a terrible disease caused by the molecular pathways that govern different biochemical processes. Oncogenic cells divide uncontrollably abnormally and attack surrounding tissues/organs [20]. Tumors have the ability to grow much faster and spread over the around tissues. For this reason, they need the elevated energy source that they encounter by forming new blood vessels during angiogenesis [84]. The main treatment modalities for cancer are chemotherapy, radiotherapy and surgery. Anti-cancer chemicals utilized treatment frequently have critical side effects [20]. Whether anticancer synthetic or semi-synthetic, agents demolish cancer cells by arresting their growth or proliferation at some point in their life cycle. These agents frequently impress non-target cellular pathways, thereby disturbing the growth of health cells. To overcome these harmful effects, the trend toward medicinal products has increased. Plant derived compounds that can prevent the carcinogenic process offer a better option to obtain effective anti-cancer therapeutic agents [94]. The tendency to discover plant-borne anti-cancer drugs has began in the 1950s with the exploration of vinblastine, vincristine, and vinca alkaloids such as isolation [34].

Derived from taxol, vincristine, vinblastine, topotecan, camptothecin derivatives, and podophyllotoxin, etoposide are well instances of such complexes with verified anti-cancer efficacy. Many natural agents such as resveratrol, silvestrol and betulinic acid with hopeful antineoplastic effects are anyway at distinct stages of clinical studies. Above all, there is direct medical practice of plant components that move as chemical patterns or templates for the design, synthesis and semi-synthesis of new matters that have the potential to prevent cancer improvement and advancement [20]

These scholarly developments have launched the United States National Cancer Institute (NCI), a comprehensive plant collection program in the 1960s that mainly focused on the mild zones of the world. These endeavors have led to the availability of numerous novel drugs with high cytotoxic effects such as camptothecins and taxanes [34]. At the same time, the improvement of these drugs to clinically effective medicinal agents has taken about 30 years from 1960 to 1990. This comprehensive herb collection program has ended in 1982, but the outbreak of novel scanning technologies has encouraged further research of novel anticancer herbal agents in 1986. This once, herbal research has extend to tropical and subtropical zones. Due to the harmful effects of synthetic anti-tumor agents, investigators around the world are currently moving toward the herbal approach to cancer treatment [20].

### Saffron

Saffron obtained by drying the *Crocus sativa* flower is known as a spice and its usage areas in conventional medicine have gone back about 3000 years. Saffron, a perennial bulbous plant of the *Iridaceae* family, is hallmarked by its unique aroma [2].

Saffron obtained from *Crocus sativa* flower is known for its anticancer properties. The effects of *Crocus sativa*'s ethanolic extract have been investigated on modulating lung cancer, skin cancer, pancreatic cancer, breast cancer and colorectal cancer in humans [20]. Saffron has been employed in another study to prevent skin cancer in mice. Novel study has also indicated that this popular spice has a strong anti-chemo-effect against liver cancer in animal testing. Biomedical findings have also showed that saffron and its main components may be beneficial against neural degenerative disorders, age-related macular degeneration, coronary artery disease, blood pressure abnormalities, acute and/or chronic inflammatory disease, mild to moderate depression, seizure, parkinsonism [88]. Amin et al. [9] have demonstrated the proapoptotic and anti-inflammatory effects of ethanolic extract of saffron against hepatocellular carcinoma both in vitro and in vivo. In addition, they have studied the antiproliferative and pro-apoptotic effects of ethanolic extract of saffron on the p53 tumor suppressor gene against different colorectal cancer cell lines. P53 gene mutation is present in 50% of colorectal cancers. Saffron extract has showed p53-dependent anticancer effects against colorectal cancer cell lines (HCT116). Saffron has triggered a large number of apoptotic pathways in these cell lines. Apoptosis, that is, programmed cell death, is a cell death mechanism in which cancerous cells escape. Tavakkol-Afshari et al. [14, 103] have used 96% ethanol saffron extract against epithelial-like human hepatocellular cells (HepG-2) and HeLa cells and observed their cytotoxic effects. However, saffron extract has not shown toxic effect against L929 healthy cell lines. Saffron includes a carotenoid compound called crocetin. The outcomes of both in vivo and in vitro studies have indicated that this compound has the potency to be a robust anti-tumor agent [88]. Saffron spice belongs to the carotenoid family, it also includes bioactive ingredients such as picrocrocin and crocin. Puglia et al. [91] have studied solid lipid nanoparticles carrying crocin and crocetin from saffron molecules on human melanoma A375 and malignant Schwann sNF96.2 cell lines and have identified antiproliferative effects. Mary et al. [76] have triggered apoptosis through mitochondria-mediated pathway, by applying crocin-conjugated PEG SeNPs against A549 cells (human lung cancer cells). They have also significantly inhibited tumor proliferation in the nude mouse model. In another study, the cytotoxic and antiproliferative effects of saffron extract and crocetin, a carotenoid from saffron, have been evaluated on A549, MCF-7 and HeLa human cancer cells and compared to non-malignant HUVECs. Saffron extract and crocetin have been shown to inhibit cancer cell growth in a concentration and time-dependent manner as well as increased cancer cell death [31]. In a study by Hoshyar et al. [36], crocin has triggered apoptosis by increasing caspase and bax activation. Crocin has revealed antiproliferative effects by stopping cell cycle progression in prostate cancer cells and inducing apoptosis in prostate cancer [19]. Crocin liposomal nanoform has been examined in MCF7 and Hela cell lines and it has been observed to cytotoxic effects by triggering apoptosis. In addition, it has been determined that it has not toxic effects on L929 healthy cells [78]. In a study by Neshaburinezad et al. [80] poly (Lactic-co-glycolic acid) -crocetin nanoparticles (PLGA-Crt NP) have been evaluated MRP1 and MRP2 activity in human ovarian cisplatin-resistant carcinoma cell line (A2780-RCIS) and parent form (A2780-RCIS). Encapsulation of crocetin PLGA NPs has enhanced the inhibitory effects on drug resistance by down-regulating MRP2 carriers. In another similar study, cytotoxicity of crocetin and crocetin encapsulated PLGA NPs has been tested in the MCF-7 cell line. Encapsulation of crocetin into PLGA substantially has enhanced the cytotoxicity and apoptotic influences of this compound. This study has shown that PLGA-crocetin NPs can be a potency therapeutic vehicle in the treatment of breast cancer [32]. Langroodi et al. [63] have investigated the efficacy of PLGA NPs contain doxorubicin and crocetin on MCF-7 cells. PLGA NPs including doxorubicin and crocetin have been found anticancer potential. Findings show that nanostructures of saffron components (crocin, crocetin and safranal) have been implemented so far, containing polymeric carriers [chitosan (Cs), sodium alginate (Alga), lactic-co-glycolic acid (PLGA)], lipidic carriers. However, more investigative is required to incorporate these findings into clinical utilizations [77].

### Lavender

*Lavender x intermedia* is a species from the *Lamiaceae* family and contains numerous molecules such as uric acid, oleanolic acid and betulinic acid. The principal components of essential oils of these herbs contain lavandulyl acetate (15.9%), alpha-terpineol (6.7%) and geranyl acetate (5%), except linalool (32.8%) and linalyl acetate (17.6%). *Lavender x intermedia* essential oils have been observed to induce cytotoxic effects in MCF7 breast cancer cells. At the same time, cytotoxic effect of *Lavender x intermedia* used with taxol has increased [101]. In vitro antitumor activities of *Lavandula angustifolia* have been examined on human prostate cancer PC-3 and DU145 cell lines. Lavender essential oil, linalool and linalyl acetate have demonstrated more much effect on PC-3 cells than DU145 cells. In the xenograft model with PC-3 cell transplantation, essential oil and linalool have importantly prevented cancer proliferation. Each of the 3 phytomolecules have importantly triggered apoptosis compared to the control group [115]. The medicinal characteristics of essential oils obtained from aromatic plants have been recognized for a long time. Currently, technological innovations have enabled old plant knowledge to lead to the identification and extraction of chemical components found in re-essential oils. These components belong fundamentally to the terpene group and are responsible for a broad range of bioactive properties ascribed to essential oils. Called 3,7-dimethyl-1,6-octadiene-3-ol, Linalool (C10H18O) is a broadly present monoterpene alcohol as the main component of plant essential oils, generally lavender and coriander. By itself, linalool is non-toxic and has been shown to have wide spread bioactive properties that would be used for pharmaceutical and cosmetic applications, with respect to recent in vitro and in vivo researchments. *Lavandula angustofolia* essential oil has been shown to be in vitro to decrease cell viability of human cervical carcinoma cells (HeLa) and lung adenocarcinoma cells (A549) [81]. In another study, *Lavandula angustifolia* has cytotoxic and apoptotic effects on HeLa and MCF-7 cell lines and apoptosis has been suggested as a possible mechanism of action [104]. Lavender aqueous extract has demonstrated a significant effect on cell proliferation and inhibited growth by necrosis. This effect has been accompanied by significant changes in protein expression of gastric tumor cells. Finally, lavender extract has been suggested as an anticancer drug candidate for further research [114]. The crude ethanolic extract of *Lavandula dentata* has displayed promising cytotoxic activity on MCF7 breast cancer cells. The conclusions of this work are the foundation for further research of *Lavandula dentata* for the potential identification of the use of new bioactive compounds with therapeutic and anticancer properties [6]. Potential anticancer and antiproliferative activities of essential oils from *Lavandula angustifolia* have been investigated by induction of both apoptosis and necrosis. To evaluate the anticancer activities of lavender essential oils, different analyzes have been performed against three cancer cell lines: A549 (human lung carcinoma), H1299 (without small lung cancer), C6 (glioma) and non-tumor HUVEC cells. Lavender essential oils have shown remarkable anticancer and antiproliferative activities against cancer cell lines by inducing both apoptosis and necrosis, both time and dose, even at low concentration and minimum exposure times. Although there is an important information about the pharmacological properties of lavender essential oils, there has been no study comparing the anticancer potentials of essential oils from first and year distillation from harvesting lavender grown under the traditional system and plastic mulch. According to literatures, the results presented in this study may be the first report conducted to investigate the in vitro anticancer and antiproliferative activities of lavender essential oils, as well as potential lysosomal and lactate dehydrogenase release. However, these results from the research presented may be useful to support the use of lavender-distilled essential oils as a promising anticancer agent in cancer treatment, as well as in vivo studies to identify mechanisms underlying anti-cancer effects [30]. Another study has shown that nanoparticles (LdAuNPs) produced with lavender have great potential to reduce K-562 cell viability, a myelogenous leukemia cell line [45].

### Clove

Cloves are *Syzygium aromaticum*, *Caryophyllus aromaticus*, *Caryophyllus silvestris*, *Eugenia caryophyllus*, *Jambosa caryophyllus* and *Myrtus caryophyllus*. The essential oil of *Syzygium aromaticum* (SA) has various biological activities such as antibacterial, antifungal, herbicidal, nematicidal, antitumor and anti-inflammatory [52].

Essential oil from the dried strainer buds of SA has been used the traditional medicine, perfume industry, for dental care, burns, pain relief and gum treatment since ancient times. When used in low concentrations, it is good for infections and problems in respiratory and digestive treatment. Previous studies on SA have shown as cytotoxic, antimicrobial, chemopreventive agent, antioxidant, anti-angiogenic and anti-inflammatory effects for anticancergenic antimutagenic human fibroblasts and endothelial cells [3].

The basic compound of clove, eugenol, is utilized as an antiseptic, antibacterial, analgesic agent in conventional medicinal applications. It is currently utilized as a flavoring agent in pharmaceutical and food products and beverages. The medicinal advantages of eugenol are well known. Recently, it has been examined for various promising biological properties. It has been notified that it has participated in photochemical reactions and has insecticidal, antioxidant and anti-inflammatory activities [24]. Naturally isolated eugenol from *Eugenia caryophyllata* has been shown to induce apoptosis in human promyelocytic leukemia cells (HL-60) [112]. Eugenol's pro-apoptotic activity has been notified in HL-60, melanoma cell line (G361) and human osteosarcoma (HOS) cells [86]. Kumar et al.[60] have researched the anticancer potential of ethanol extract and clove essential oils for MCF-7 human breast cancer cells. Lesgards et al. [68] have notified that clove essential oil consisted of phenylpropanoids and terpenoids with antitumor activity in tumors both in cell lines and in animals. Dwivedi et al. [24] have researched the comparative anticancer potential of oil, ethanol and water extract against DU-145 cells, breast cancer MCF-7, (ER+) with prostate cancer, HeLa cervical cancer, TE-13 esophageal cancer, MDA-MB-231 (ER− and) and normal human peripheral blood lymphocytes. Clove oil has showed maximum cytotoxic activity on TE-13 cells and maximum cell deaths (80%) within 24 h. On the contrary, it has been observed minimum cell death in DU-145 cells, but not cell death in human peripheral blood mononuclear cells (PBMCs) in the same dose. Banerjee et al. [16] have showed the chemopreventive potential of aqueous infusion of cloves for lung cancer in type A mice. Clove infusion has significantly reduced the count of proliferating cells and the count of apoptotic cells. Kouidhi et al. [58] have investigated the cytotoxic and anticancer activity of clove essential oil on normal cells (MRC-5) and cancer cells (A549, raw 269.7, HT29 and Hep2). Raghunandan et al. [93] have notified that functionalized gold nanoparticles developed with clove bud extract have inhibited 50% of proliferation of HeLa cancer cells at a dose of 20 µg/mL after 48 h of incubation. Vivek et al. [106] have obtained a similar cytotoxicity report at a concentration of 30 µg/mL (IC50). Shanthi et al. [99] have synthesized palladium nanoparticles with *Syzygium aromaticum* and the synthesized PdNPs have caused a concentration-dependent inhibition against HeLA cancer cells, which may be due to pharmacologically active compounds retained on the surface of clove buds. In another study, clove extract has increased cytotoxic effects of gemcitabine on Hela cells [37]. Clove extracts mediated FMSP-nanoparticles have led to cancer cells death in MCF7 breast cancer cells [54]. *Syzygium aromaticum* has increased the cytotoxicity of tamoxifen against MCF-7 at low concentration of the drug, but it has been a strong antagonistic effect at high concentration [3]. Jaganathan et al. [39] have showed the molecular mechanism of eugenol-induced apoptosis in human colon cancer cells. Li et al. [69] have found that aqueous extract of clove has inhibited tumor growth by inducing autophagy through AMPK/ULK pathway on human pancreatic ASPC-1 and human colon HT-29 cancer cells. Two fractions of clove have induced typical apoptosis in human non-small cell lung cancer cell line H1299. Chloroform extract of clove bud has been used to treat lung cancer [8]. Clove and ethyl acetate extract (EAEC) have been investigated for in vitro and in vivo antitumor effects. Clove ethyl acetate extract has demonstrated cytotoxic effects against several human cancer cell lines. The in vivo effect of EAEC has been researched using the HT-29 tumor xenograft model. Both EAEC and oleanolic acid have showed cytotoxic activities including several human cancer cell lines (SKOV-3, HeLa, BEL-7402, HT -29, MCF-7) [71].

Lately, the biological activities of the kumatakenin, a flavonoid isolated from cloves, have been characterized poorly. Anticancer effects of the kumatakenin have been researched on human ovarian cancer cells and tumor-associated macrophages (TAM). The kumatakenin has been shown to exhibit significant cytotoxic activity in human ovarian cancer cells SKOV3 and A2780. The kumatakenin has been found to exhibit anticancer activities by inducing apoptosis of ovarian cancer cells and inhibiting the activation of tumor-dependent macrophages [110]. The clove bud nanoscale emulsion system, produced using varying concentrations of surfactant, has been evaluated in the thyroid cancer cell line (HTh-7). Due to the effect of clove bud oil-based emulsion, a decrease in cell viability has occurred in the thyroid cancer line [83]. Two components (oleanolic acid and eugenol) of the active fraction of clove have been found to exhibit cytotoxicity against various cancer cells. Oleanolic acid has induced apoptosis of cancer cells through the mitochondrial pathway. The combination of oleanolic acid and fluorouracil (5-FU) treatments synergistically have enhanced the cytotoxicity of 5-FU against human pancreatic cancer Pan-28 cells. The active fraction of clove has been found to be more effective against human colon cancer HT-29 xenografts in vivo than a single isolated oleanolic acid or eugenol component. The active fraction of clove has induced apoptosis and autophagy in human colorectal cancer HCT-116 cells [72]. The anticancer effect of clove buds has been proved in vitro (MCF7) and in vivo breast carcinoma model [59]. The anticancer activity of the combination of simple aromatic benzoate (SAB) compounds and eugenol (AB) has been evaluated against colon cancer cell line HCT116. Simple aromatic compounds have showed greater activity on HCT116 and WiDr cell line and combined EU-TFBA has showed more activity than combined EU-SAB against HCT116 and WiDr cell line [27]. Clove’s crude ethyl alcohol extract has suppressed the proliferation of human gastric cancer (AGS) cells, possibly due to the induction of apoptosis [51]. The anticancer effects of fennel and clove oil have been investigated on Caco-2 cells and normal human lymphocytes, and the oil mixture has applied selective cytotoxicity to human epithelial colorectal adenocarcinoma Caco-2 cells through cell cycle arrest and apoptosis [26].

### Red beet

Beet is a plant in the *Beta vulgaris Chenopodiaceae* family. It is also known for its many cultivated varieties with purple root vegetables, best known as beets or table garden beets. Beet has been utilized in conventional medicine for hundreds of years for the treatment of constipation, intestinal and knuckle pain, bran. Modern pharmacology demonstrates that red beet extracts reveal excellent antioxidant activity as well as antihypertensive and hypoglycemic activity. The promising outcomes of phytomolecules in health protection ensure the opportunity to be used in functional foods [13]. Beetroot is the 10th vegetable in the world that contains antioxidants. These antioxidants are used as cleansers of free radicals and prevent oxidative damage on proteins, DNA and lipoproteins. Oxidative damage of macromolecules can lead to chronic diseases such as cancer, cardiovascular disease, neurodegenerative diseases and stroke, which can be prevented by antioxidant compounds in beetroot. Red beet also has high concentrations of secondary metabolites (phenolic acids, flavonoids, ascorbic acid) [25].

Beetroot (*Beta vulgaris var. Rubra* L.) is a vegetable rich in carbohydrates, fat, micronutrients and components with bioactive properties. Bioactive components contain water soluble pigments of betaine, polyphenols, carotenoids, flavonoids, saponins and betalains [64]. Beetroot (*Beta vulgaris var. Rubra* L*.*) contains phytomolecules that have helpful effects on human health. Betaxanthine and betacyanine show anti-inflammatory and antiproliferative activities, as well as significant amounts of antioxidant activity [13, 82]. In these days, there is a major attention in the anticancer effects of red beet (*Beta vulgaris* L.) pigment extract, which is utilized worldwide as the red food color E162 and as a natural colorant in cosmetics and medicines [48]. Synergistic cytotoxicity of red beet (*Beta vulgaris* L.) extract with doxorubicin has been researched on human pancreatic, breast and prostate cancer cell lines [49]. In combination with vitexin-2-O-xyloside, betaxanthins and betacyanins, it has been found to exert antiproliferative activity on breast, liver, colon and bladder cancer cell lines by induction of both intrinsic and extrinsic apoptotic pathways [82]. Betanins have impressed both the tumor surroundings and the tumor cell itself. Understanding how this happens is the focal point of further research [64].

Lately, there has been a great attention in the effects of red beet root on human health. Numerous analyzes have been conducted on the antioxidant, anti-inflammatory and chemo-preventive *Beta vulgaris* phytochemical activity, its effect on the gastrointestinal and cardiovascular system, and endurance exercise performance. The act of red beet nitrates in biological transformation and blood pressure arrangement has been defined in detail. *Beta vulgaris* phytomolecules have ensured an anti-proliferative effect on breast, liver, colon and bladder cancer cell lines by induction of both intrinsic and extrinsic apoptotic pathways [82]. Lee et al. [66] have reported on betanin and showed in vitro anti-proliferative effects against HepG2 cancer cell lines. The cytotoxic effect of beet extract has been compared to doxorubicin (adriamycin) in human prostate (PC-3) and breast cancer (MCF7) cell lines [47].

*Beta vulgaris* (beet) root extract has demonstrated an in vivo anti-tumor promoting activity against the skin of mice [50]. Drinking water with beetroot food color has antagonized esophageal carcinogenesis in rats treated with N-nitrosomethylbenzylamine [65]. Numerous researchments have been conducted to research the anticancer effects of betalains of beetroot extracts against human tumor cell lines such as breast, lung, kidney, stomach, prostate and colon cells [17]. Betalains from beets have acted by reducing colon cancer cell proliferation and have induced apoptosis without showing cytotoxicity [46]. The betacyanin-rich extract has not showed cytotoxicity to two normal human cell lines at the doses tested, in vivo studies using animal models for further verification are still required [48, 111]. The percentage of viability of the lung cancer cell line (A549) has been reduced by increasing the doses of the red beet's methanolic extract. Conversely, the viability of colorectal adenocarcinoma Caco-2 has been not impressed by red beet root doses, except for high doses (800 μg/ml), which showed a mild decrease in the viability of the Caco-2 cell line [25]. *Beta vulgaris* extract-mediated biosynthesized silver nanoparticles have showed cytotoxic effects against MCF-7, A549 and Hep2 cells compared to normal cell lines [105].

### Nanoforms for anticancer plant drug distribution

Recently, nanotype has been confirmed by the US Food and Drug Administration (FDA) for human use, and some are undergoing clinical trials [55]. The improving of nanoscience has been a benefaction for humanity, has paved the way for various applications in therapeutics [99]. Nanoparticles have indicated some exciting outcomes in cancer cells due to their specific targeting, biocompatibility, bioavailability and multifunctional abilities. A few researches have indicated that nanoparticles have anticancer effects when analyzed under in vitro and in vivo terms [56]. With the success of these compounds improved as leading drugs for cancer treatment, novel technologies are arising to further improve the field. Novel technologies contain nano-drugs aimed at increasing the anticancer activities of plant-derived drugs, controlling the release of the compound, and investigating novel methods for application [88].

Cancer is stated to be a important health problem in the twenty first century. The status is even more difficult when it comes to treatment using chemotherapy, which uses synthetic anticancer molecules with numerous side effects. Lately, there has been a paradigm shift toward the adoption of herbal medicines for cancer treatment. In this context, an appropriate delivery system is essential for the delivery of these herbal biomolecules, especially to the tumor area. To succeed this aim, carbon nanotubes (CNTs) have been extensively researched to distribute anticancer herbal molecules with developed treatment efficacy and safety [43]. Lately, many multifunctional delivery systems have been synthesized to use different agents, including micelles, liposomes and inorganic nanoparticles. PLGA (poly-D, L lactide-co glycolide) is one of the ideal candidates in the synthesized of multifunctional delivery systems and has been confirmed by the FDA for the synthesized of drug delivery systems. Compared to other carriers such as liposomes, PLGA NPs are an advanced tool in drug delivery due to their unique properties including biocompatibility, bioavailability, high drug loading capacity, stability and sustained drug release [54].

Nano-scale drug delivery systems are important tools for improving pharmacokinetics and bioavailability of drugs and natural active compounds [91]. All pieces of a herb that contain antioxidants or sugars, including leaves, fruits, roots, seeds and stems can be utilized in the nanoparticle synthesis process, replacing potentially dangerous chemicals. This is because herb extracts work very great in the synthesis of nanoparticles [99].

Nanoparticles have some side effects or toxic effects at high doses, but this issue may be deciphered if the nanoparticles are handled with other herbal extracts. For a few years, combination therapy has been one of the most promising improvements to succeed high therapeutic effects with very low toxicity. One of the substantial strategies to increase the effectiveness of nanoparticles is to combine low-dose nanoparticles with drug or herbal extracts. Moreover, combination therapy has an important role in reducing drug resistance, hazardous effects and chemoresistance, which is a crucial problem in cancer treatment [54].

Apart from these, graphene/graphene oxide, chitosan, solid lipid nanoparticle and nanoemulsion designs have been the most preferred nano-carrier materials in recent years due to their high biocompatibility and easy to manipulate [40, 75, 85].

### Graphene oxide and its anticancer effects

Graphene is of great interest due to its extremely outstanding mechanical, electronic, optical and thermal properties. Owing to its unique two-dimensional sp2 bonded carbon networks, it serves as an extremely significant material for many implementations such as energy storage tools, flexible electronics, sensors and solar cells. Various approaches such as micromechanics, reduction of graphene oxide (GO), epitaxial growth and chemical vapor deposition have been used in graphene synthesis. Among these methods, the reduction of GO has demonstrated significant advantages in terms of efficiency and cost compared to another methods and is therefore considered the most effective way to meet the requirements of large-scale graphene application. Graphene also has superior biological properties such as drug release, antibacterial agents, biomolecule detection, cellular imaging, and anticancer activity. The improvement of nanocomposites, which include the combination of carbon nanomaterials with nobel metal nanoparticles, will significantly increase anticancer effect due to its unique physicochemical properties, higher surface area and stronger inhibitory effect [53, 67].

In a novel study by Russier et al. [21, 97], a newly explored form of graphene called "several layer graphene" (FLG) has been used to treat myelomonocytic leukemia. FLG has been notified to have a specific effect on cancerous cells without any visible side effects. A mostly utilized polymer that interacts with GO is chitosan (CS), which has mucosal adhesiveness, biodegradability, antibacterial activity, low immunogenicity, a polyelectrolyte nature and solubility in various media. Synthesis of chitosan-functionalized graphene oxide has been obtained as a nanocomposite to yield grape seed extract rich in flavonoids, and to evaluate cytotoxicity on a human kidney cell line. It has been found that chitosan-graphene oxide-extract complexes are not toxic to kidney cells compared to the crude extract at the concentrations studied. This nanocomposite has been expressed to could be utilized as a novel phyto-drug delivery carrier [29]. In another study, camptothecin-loaded graphene oxide nanoparticle and functional polyethylene glycol and folic acid nanocomposite have been examined on breast cancer cell lines (MCF-7) and have showed high cytotoxicity against cell lines [21]. The combination of graphene oxide-silver nanoparticle nanocomposites and cisplatin have increased apoptosis and autophagy in human cervical cancer cells [113]. 5-Fluorouracil and curcumin co-encapsulated chitosan/reduced graphene oxide nanocomposites have caused cytotoxic effects against human colon cancer cell lines [22]. Gold nanoparticles reduced graphene oxide with hybrid nanocomposite curcumin cap have been shown to have antioxidant potential and selective cancer cytotoxicity [5]. According to in vitro biological studies, normal fibroblast (3T3) and liver cancer cells (HepG2) have been treated with graphene oxide (GO), GOGA nanocomposite, and gallic acid (GA) for 72 h, and GOGA nanocomposite has indicated anti-cancer effect without affecting normal cell growth [23]. Curcumin and paclitaxel overloaded on polymerized reduced graphene oxide with the function of MNA-MB-231 have indicated a high-effect synergistic anticancer effect on breast cancer and A549 lung cancer cells [79]. Graphene Oxide/Chitosan Oligosaccharide/γ-Polyglutamic Acid Composites have showed significant cytotoxicity and apoptotic effects against Hela cells [70]. In an another work, *Ganoderma lucidum* (GL) has been used as a stabilizing agent of the graphene-Fe_3_O_4_ composite and used as a drug carrier targeted for lung cancer. The synthesized nanocomposites have clearly showed cytotoxicity against A549 cancer cells by the action of GL due to its anticancer properties and have a high potential to be a targeted drug carrier for cancer therapeutics. Nanocomposites containing GL have exhibited a higher cytotoxic activity than nanocomposites without GL [67]. In the light of these findings, It can be said that graphene oxide could be an important factor in the formation of nanoforms of naturally originated phytomolecules, and that the investigation of anticancer efficacy would guide further cancer studies.

### Solid lipid nanoparticles and anticancer activity

Graphene/Graphene oxide nanomaterials are preferred over nanocarts as they are available in a wide variety of carrier designs. However, solid lipid nano-carriers (SLNs) are currently the most promising and new lipophilic drug carriers [73]. SLNs are used by researchers for drug delivery and drug substitute colloidal with global morphology. The average size of 5 SLN is between 150 and 300 nm, but can reach up to 1000 nm with respect to the surfactant. SLNs have various advantages such as low or no toxic effect on healthy tissue and ease of production in larger production units, the ability to load both lipophilic and hydrophilic therapeutic agents, and high drug load capacity [15, 85]. Solid lipid nanoparticles (SLNs) are nanoscale drug delivery systems (DDSs) consisting of a solid lipid. These nanostructures are usually balanced with surfactants. It also dissolves drug molecules. SLNs show less toxicity and offer easy cost-effective large-scale production using high-pressure homogenization [15]. In one study, sclareol-loaded solid lipid nanoparticles have showed anti-proliferative effects on A549 human lung epithelial cancer cells. Flow cytometry analysis has detected early and late apoptosis in cells treated with sclareol and sclareol-SLNs [35]. Omega-3 PUFA loaded on resveratrol-based solid lipid nanoparticles have caused antineoplastic activities in human colorectal cancer cells [98].

Encapsulation of linalool, a monoterpene found in the essential oils of plants in solid lipid nanoparticles, has shown in vitro antiproliferative effects on hepatocarcinoma (HepG2) and lung adenocarcinoma (A549) in a dose-dependent response and higher inhibitory effects compared to free linalool [96]. Bombesin conjugated solid lipid nanoparticles loaded epigallocatechin-gallate have reduced tumor volume and survival for treated tumor-bearing mice [92]. Resveratrol-loaded solid lipid nanoparticles (Res-SLN) have displayed more potent inhibitory effects on the infestation and migration of MDA-MB-231 cells. Res-SLN has been shown to have major potency for breast cancer treatment [109]. In triple negative breast cancer cells, curcumin-loaded solid lipid nanoparticles have eliminated p-glycoprotein-mediated doxorubicin [28]. In the light of these findings, it can be said that solid lipid nanoparticles would be an important factor in the formation of nanoforms of naturally originated phytomolecules, and that the investigation of anticancer efficacy would guide further cancer studies.

### Phytomolecules and Covid-19

The SARS-CoV-2 (Severe acute respiratory syndrome coronavirus 2) genome codes distinct structural and non-structural proteins. The viral spike (S) protein is the basic constructional protein in the process of cell invasion, as it facilitates the interplay of the host cell with angiotensin converting enzyme 2 (ACE2). Because it is mediated by the host cell transmembrane protease serine 2 (TMPRSS2). These constituents could function as molecular inhibitors to disable the ability of virus to enter the cell. Moreover, the unstructured parent protease (Mpro) eases proteolytic processing of polyproteins and hence makes it an interesting target for drug design research by controlling viral gene expression and replication processes [4]. Based on the molecular docking study, the *Syzygium Aromaticum* plant with such a compound has been found to show better and significant binding energy against these receptors [44]. Many drugs with promising results such as Hydroxychloroquine and Azithromycin as well as Chloroquine phosphate, Remdesivir are currently being tested in clinical trials. On the other hand, herbal extracts and substances used in viruses such as SARS and H1N1 could be used as an alternative approach in the treatment of COVID-19. The variety in medicinal plants and their biomolecules have required a rapid assessment of probable viral inhibitory activity, which may initially be supported by ligand binding simulations [4]. In one study, it has been demonstrated by molecular docking studies that the crocin molecule of *Crocus sativus* saffron plant is the main inhibitor of SARS-CoV-2. The synthesis of this molecule and its evaluation of in vitro and in vivo activities against the main protease of SARS-Cov-2 have been thought to could be interesting before clinical trial [1]. Among the promising compounds for coronavirus inhibition in humans are lectins such as scutellarein, silvestrol, saikosaponin B2, quercetin, myricetin, caffeic acid, psoralidin, isobavacalcone and griffithsin. Other compounds, such as lycin, may be appropriate if a therapeutic level of antiviral activity can be obtained without exceeding toxic plasma concentrations. It has been reported that most of promising molecules identified as coronavirus inhibitors are classified as polyphenols and include a conjugated fused ring structure [74].

### Antiviral effective nanocomposites

#### Graphene oxide and Covid-19 (SARS-CoV-2)

Basic studies of nano-bio interactions can be adapted to understand how SARS-CoV-2 affects its cells (eg, SARS-CoV-2 is 60–140 nm and angiotensin converting enzyme receptor 2 binds to ACE2). It could be lead to the new therapeutic agents and design [18]. The new coronavirus (SARS-CoV-2) has been initially explored and spread in Wuhan, China in late 2019, and since then, this coronavirus-induced disease has been reported by the world healthcare organization (WHO) as coronavirus disease 2019 (COVID-19). The contagion factors for the disease are still contentious; nevertheless, the new virus reservoir is thought to be bats, it contains a wide variety of coronavirus variations and is therefore considered to be the natural reservoirs of SARS-like coronaviruses. On the other hand, the contagious host that the virus initially infected to humans is yet unknown [33]. In 2019, pneumonia coronavirus disease outbreak has been a global issue. As government bodies have struggled to prevent anymore extend of COVID-19, investigators have occasionally launched experiments on vaccines and a clinical experiments are now underway with potential treatments for severe acute respiratory syndrome coronavirus-2 (SARS-CoV-2). The coronavirus is created by surface protein projections on the viral lipid envelope surrounding the single-stranded positive-sensing RNA. Two-dimensional material graphene has attracted great attention due to its promising antimicrobial applications and also showed antiviral efficacy. The first proof of graphene antiviral effects has been notified in 2012 when thin films of rGO-tungsten oxide have been used for photoinactivation of bacteriophages under visible light irradiation. Functional graphene can capture viruses and can be given as an antiviral drug. It has recently been shown how the SARS-CoV-2 Spike S1 protein receptor binding domain can interact with heparin and change conformation. This has implications for primary care therapeutic development by repositioning heparin and glycosamminoglycan-based antiviral including sulfated GO derivatives [87].

#### Anticancer and antiviral potential of nano-sized emulsions

Recently, nanoemulsions (NEs) have taken great interest due to their broad feasibility in drugs and other industries. Nano-sized emulsions ensure many benefits due to increased surface area and thence evident effects on bioavailability, and they can be utilized as a new drug delivery system and can be used in place of liposome and vesicle. Besides, NEs maintain active ingredients against physicochemical stress and prolong continuity compared to unrestricted medications, and facilitate additional routes such as oral, tropical and intravenous administration. In addition, the solubility of lipophilic compounds can be developed in the form of an emulsion, which increases their bioavailability and pharmacokinetic properties in water [55, 56]. Nanoemulsion is an emulsion system occuring of oil, surfactant and water with a particle size of less than 200 nm and isotropic appearance [75].

The nanoemulsion system has demonstrated compatibility with the requirement for pulmonary delivery application. For instance, high inhibition of the lung cancer cell of docetaxel-loaded nanoemulsion (50% reduction in A549 when exposed to 76.41 μg/mL) has been reported. In general, the nanoemulsion drug protects against deterioration and UV light. Other advantages of nanoemulsion contain ease of preparation, stability of thermodynamics and increased surface area due to fine particle size. Nanoemulsions show the potential to deliver active compounds to the lungs, thanks to their high drug loading efficiency. (1.5 mg/mL budesonide; 0.05% quercetin in nanoemulsion). Also, it can improve pulmonary accumulation and involvement, causes prolonged periods in lung tissues [107]. Nanoemulsion technology is broadly utilized in the nutrient and pharmaceutical industries as safe efficient delivery systems. Nanoemulsion drug delivery systems are successfully employed as a high performance curative strategy in cancer treatment. Nanoemulsions as new biomedical tools make it possible to effectively treat breast cancer. Nanoemulsion of cherry kernel oil has been investigated cytotoxic, apoptotic and anti-tumor activity in MCF7 cells and breast cancer mouse model (vaccinated with TUBO cells). The results have showed that apoptotic death induction of 36.5 nm stable cherry oil nanoemulsion has importantly reduced viability of MCF7 breast cancer cells compared to normal healthy HFF cells and reduced tumor size in the murine model [107]. In another study, carvacrol nanoemulsion has induced the production of reactive oxygen species (ROS) in A549 lung cancer cells, leading to activation of key regulators of apoptosis such as p-JNK, Bax and Bcl2, and activation of the caspase cascade. The outcomes have strongly supported that ROS induction has induced apoptosis in A549 cells [56]. In another similar study, carvacrol nanoemulsion has induced cell cycle arrest, apoptosis induction and autophagy inhibition in the doxorubicin-resistant A549 cell line [55]. Wahgiman et al. [107] have evaluated nanoemulsion containing gemcitabine against human fetal lung fibroblast (MRC5) and human lung carcinoma (A549) cells. It has been found to be less toxic to MRC5 healthy cells. Tao et al. [102] have produced *Ginkgo biloba* leaves polyprenol and Fullerene C60 nanoemulsion with folic acid bound chitosan and graphene oxide nanocomposites (NCs). This nanoform has effectively prevented the overexpression of Akt1 and Akt2 against MHCC97H cells and significantly increased PTEN expression.

In a study, *Ginkgo biloba* L.'s nanoemulsion has been tested for growth inhibition against influenza A and Hepatitis B viruses, direct killing the virus, and MTT bioactive activities in MDCK and HepG 2215 cells infected with the H3N2 virus. According to the nanoemulsion results of *Ginkgo biloba* L., it has been expressed that it could have significant roles in improving the mobility, stability and permeability of the cell membrane, strengthening membrane fusion and regulating the structure and function of biological membranes [108]. Sweeney et al. [100] have synthesized Poly (lactic-co-glycolic acid) PLGA with a nanoemulsion system to encapsulate both prostratin protein and anti-CD25 using nanodepots prostratin-antiCD25-nanodepots, and have administered using J-Lat 10.6 cells in an in vitro model of latent HIV and acute T cell leukemia. Compared to prostratin plus anti-CD25 and other controls, natural killer cells (NK) have mediated cytotoxicity, which are effector cells of the immune system, significantly increased. These findings demonstrate the applicability of prostratin-antiCD25-nanodepots to target cells to increase the cytotoxicity of NK cells as antiviral or antitumor agents. In the light of these findings, nanoemulsions could be an important factor in the formation of nanoforms of naturally occurring phytomolecules, and that in vitro and in vivo investigation of anticancer-antiviral activity would guide further clinical studies.

## Conclusions

Medicinal and aromatic plants are native crude substances. Since past times, these plant substances are being generally utilized as herbal drugs, nutrient products, and cosmetics. Phytomolecules derived from medicinal and aromatic plants are in great demand, especially in the pharmaceutical industries. In the other hand, these phytomolecules have certain limitations such as low absorption, high toxicity and other side effects, bioavailability and efficacy. These limitations can be overcome by using nanotechnological vehicles. Herbal extracts or essential oils are also helpful in the production of nanoparticles. In the near future, this combinatorial application of medicinal and aromatic plants and nanotechnology will be advantageous in the field of health [61]. Cancer is a terrible ailment and represents one of the biggest health problems for the human race and requires a proactive strategy for treatment. Herbs are sources for new chemical formations and ensure promising applications for cancer studies [38].

Researchers from all over the world have focused on searching for new drugs, paying special attention to the application of antitumor compounds from natural sources. If we look at the history of treatments against cancer, most of them targeted "cancer symptoms", tumor-specific changes have been created and described by Hanahan and Weinberg as follows: maintaining proliferative signaling, avoiding growth suppressors, resisting cell death, ensuring reproductive immortality, inducing angiogenesis, invasion and metastasis to activate, preventing immune destruction, causing cancer-causing inflammation, genome instability and mutation, and releasing cellular energy. In particular, recent evidences about the molecular mechanisms of carcinogenesis emphasize the significance of genetic and epigenetic changes as an significant subject in cancer prevention and treatment. The significant effect of natural compounds on epigenome has been notified in relation to carcinogenesis-related processes such as histone modifications (methylation, acetylation and phosphorylation) associated with changes in chromatin structure as well as DNA methylation and non-encoding microRNA expression [90]. Consequently, these modifications have a wide impact on the expression of target genes, among others, oncogenes and tumor suppression genes, thereby affecting cancer initiation or progression. According to recent research, in the next decade, it is important to apply compounds of natural origin that exhibit deep molecular and epigenetic activity in the clinical cancer treatment routine. If we examine the substances of natural origin whose anticancer activity is well known below:

1. *Mitotic inhibitors* Vinca alkaloids—Vinblastine, Vincristine, Vindesine, Vinorelbine, Vinflunine, Colchicine, Podophyllotoxin, Taxanes (Paclitaxel, Docetaxel).
2. *I and II topoisomerase inhibitors* Camptothecin, Topotecan, Irinotecan, Etoposide
3. *Inducers of xenobiotic metabolism* Allyl sulfide, Indole-3-carbinol, Phenethyl isothiocyanate, Sulforaphane, Iberin Terpenes Coumarins.
4. *Polyphenols* Carnosol, Resveratrol, 6-gingerol, Honokiol, Flavonoids [90].

It is value emphasizing again that most of the cytostatics confirmed for medical treatment are substances of natural origin, analogues and metabolites. Owing to its non-selective activities and hazardous effects, there is still a robust necessity to investigation and improve novel drugs and ingredients of natural origin to remedy human cancer therapy. The above-mentioned substances offer both low toxicity and potential selectivity against cancer cells. Unlike chemotherapeutics used in present cancer therapy, they can be tolerated even in high doses within the human organism. Some of these compounds can be utilized in photodynamic therapy or reversal of multiple drug resistance. Furthermore, some researches show that molecules from medicinal plant prevent carcinogenesis reducing the risk of death and prolonging survival among cancer patients. Considering all these and considering the process of increasing biodistribution, the use of substances by electroporation, sonoporation or encapsulation in special nano-carriers could be an interesting alternative to classical therapy [1]. Each year numerous nano-drugs have gone into clinical trials and about 56 clinical trials, including the term "nano". Not only do phytomolecules transport, but medicinal and aromatic plants are useful in the synthesis of a variety of metallic nanoparticles. The long-term toxicity, side effects, low solubility and low stability of the pharmaceutically active compounds of medicinal and aromatic herbs have become a major problem, and led to the demand for a new drug delivery system that can reduce or completely eliminate the effect associated with the active compounds [61].

This review highlights that nanotechnology and the nanocompositions of medicinal and aromatic plants would be a useful approach for development of further anticancer and antiviral treatments. Therefore, the nanocomposites of phytomolecules would obtain from saffron, clove, lavender and beetroot plants could offer new alternative treatments in the research of cancer and covid-19 diseases. Evaluation of in vitro and in vivo activities of these nanocomposites could be effective in obtaining important data for further clinical trials. There are hardly any work investigating of anticancer and antiviral activities of the nanoemulsion, graphene oxide and solid lipid nanoforms of these phytomolecules (crocin, crocetin, linalool, linalyl acetate, betulinic acide, eugenol, kumatakenin, betanin, betacyanin, betalains etc.)

## Data Availability

Not applicable.

## Abbreviations

ACE2: Angiotensin converting enzyme 2
Beetroot: *Beta vulgaris* Var. Rubra L.
Beet: *Beta vulgaris*
COVID-19: Coronavirus disease 2019
CS: Chitosan
EAEC: Ethyl acetate extract
FDA: Food and Drug Administration
GO: Graphene oxide
NCI: National Cancer Institute
NEs: Nanoemulsions
OA: Oleanolic acid
SARS-CoV-2: Severe acute respiratory syndrome coronavirus 2
SA: *Szygium aromaticum*
SLNs: Solid lipid nano-carriers
WHO: World Healthcare Organization
